# Large-cell neuroendocrine tumor of the prostate: a case report and review of the literature

**DOI:** 10.1186/s13256-021-02830-5

**Published:** 2021-05-07

**Authors:** Walid Sleiman, Omar Karray, Mikael Abi Abdallah, Sarah Bleichner-Perez, Jihen Kourda, Mihaela Cosma-Opris, Sabine Assouad, Jean-Charles Riffaud, Stéphane Bart, Patrick Coloby

**Affiliations:** 1 Urology Department, René Dubos Hospital, Pontoise, France; 2 Nuclear Medicine Department, René Dubos Hospital, Pontoise, France; 3 Pathology Department, René Dubos Hospital, Pontoise, France; 4 Oncology Department, René Dubos Hospital, Pontoise, France; 5 Centre de Radiothérapie et d’Oncologie Médicale de Pontoise, Osny, France

**Keywords:** Neuroendocrine carcinoma, Prostate neoplasms, Prostatectomy, Local neoplasm recurrences

## Abstract

**Background:**

Primitive neuroendocrine prostate neoplasms are rarely reported. This entity comprises carcinoïd tumors and poorly differentiated neuroendocrine tumors, mainly those of the small-cell type. Large-cell-type primitive tumors are exceptional, and only nine cases are reported in the literature. Similar to neuroendocrine tumors of the prostate, large-cell-type primitive tumors may be observed in the context of conventional adenocarcinoma during androgen deprivation therapy or as prostatic metastasis of a distant neuroendocrine tumor, mainly pulmonary neoplasms.

**Case presentation:**

We report a Caucasian case of a mixed prostatic carcinoma, with the largest component being the large-cell neuroendocine carcinoma, in a patient who underwent a total prostatectomy for a localized cancer. Diagnostic, histological, therapeutic and evolutive aspects are reported and discussed.

**Conclusions:**

Large-cell primitive prostate neuroendocrine carcinoma is a rare but aggressive histological entity, which can be associated or not with an adenocarcinomatous component. Mixed forms have a better outcome, mainly when diagnosed at an early stage.

## Background

The 2016 World Health Organization’s histological classification of prostate cancer includes well-differentiated carcinoid tumors and small- or large-cell poorly differentiated tumors within the category of neuroendocrine tumors [1]. Only 20 cases of large-cell neuroendocrine prostate tumors are described in the literature, among which are nine cases of primary tumors [2]. Large-cell neuroendocrine prostate tumors are a rare histological entity whose evolutive profile and therapeutic potential differ from those of conventional adenocarcinoma. Primary prostate neuroendocrine tumors can be pure or associated with an adenocarcinomatous component. Mixed forms have better prognosis when diagnosed early at a localized stage.

## Clinical case

A 68-year-old Caucasian patient presented in September 2015 due to an elevated prostate-specific antigen (PSA) level. Past medical history included diabetes mellitus, hypertension, dyslipidemia, hyperuricemia and mild renal failure. The PSA was 6.67 ng/ml in February 2015 and 9.65 ng/ml in September 2015. Upon clinical examination, a medium-sized prostate was determined, with a palpable induration of the left lobe, clinically classified as a stage T2b cancer. Twelve ultrasound-guided prostate core biopsies were taken in October 2015. All biopsied tissue from both lobes was found to have tumor infiltration by a large-cell neuroendocrine carcinoma associated with acinar adenocarcinoma with Gleason score 7(3 + 4), with perineural invasion and without capsule infiltration. Multiparametric magnetic resonance imaging of the prostate in December 2015 showed a prostate that weighed 43 g with a suspicious lesion in the posterior peripheral zone, between the apex and the midgland of the left lobe, classified as Pi-Rads 5/5, without infiltration of seminal vesicles and without capsular invasion or pelvic adenopathy. A bone scan in November 2015 showed no bone secondaries.

**Fig. 1:**
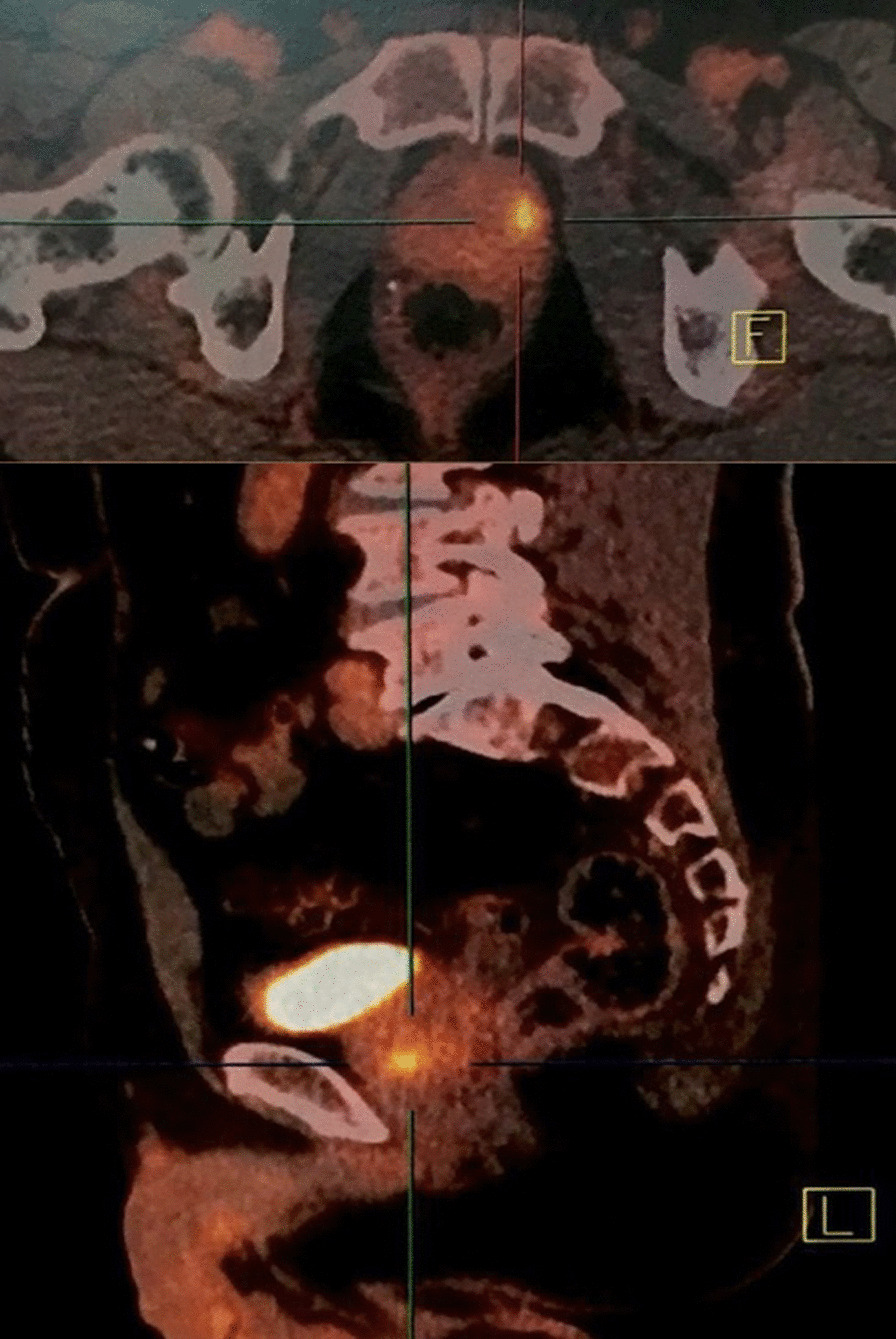
^18^F-Fluorodeoxyglucose positron emission tomography/computed tomography: isolated metabolic uptake of the left lobe of the prostate

Given that the majority component revealed on the biopsies was the neuroendocrine component, a ^18^F-fluorodeoxyglucose positron emission tomography/computed tomography ([^18^F]FDG PET/CT) was performed in November 2015, showing a prostate metabolic hyperfixation without suspicious distant fixation (Fig. 1).

Taking into consideration the rarity of the histological entity and the localized tumor character, we decided to carry out curative surgical treatment by radical prostatectomy with bilateral extended pelvic lymph node dissection. Surgery was performed in January 2016. Operative exploration revealed that the prostate tumor had invaded the bladder neck and trigone. Radical prostatectomy, bilateral pelvic lymph node dissection and partial cystectomy involving the bladder neck and trigone in macroscopically tumor-free limits and bilateral ureterovesical reimplantation were performed.

**Fig. 2:**
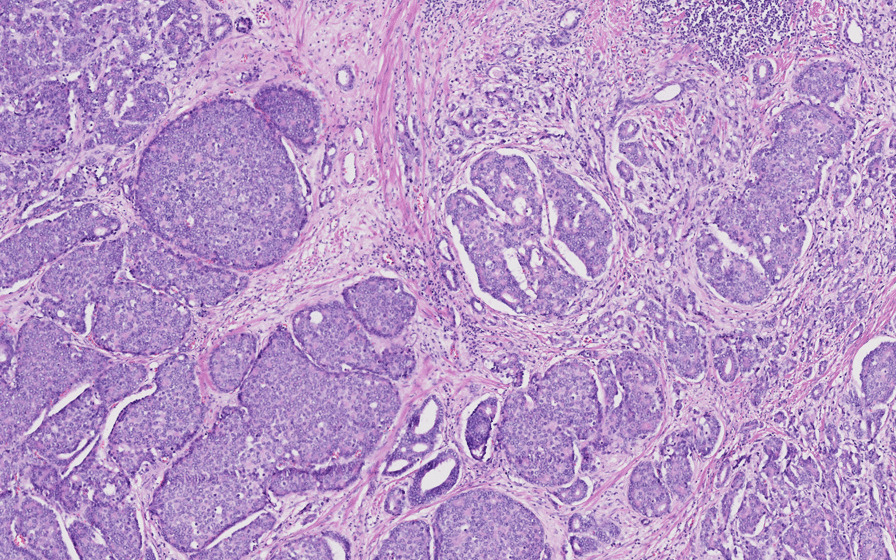
Operative specimen from total prostatectomy: hematoxylin and eosin staining. Some small adenocarcinoma glands can be seen between clusters of the large-cell neuroendocrine component

**Fig. 3:**
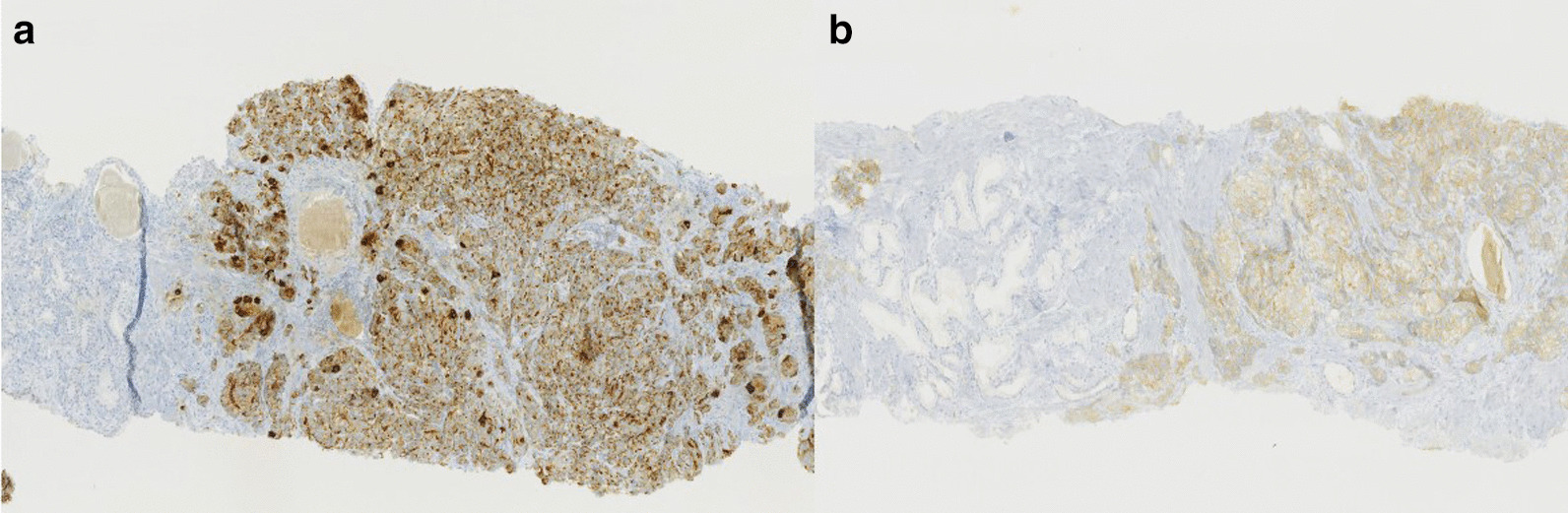
Operative specimen from total prostatectomy: immunohistochemistry. Positive labeling for chromogranin A (**a**) and synaptophysin (**b**). These two markers are positive for the neuroendocrine component and negative for the adenocarcinomatous component.

Based on histology, we was concluded that this tumor was a bilateral mixed prostate carcinoma that had invaded 80% of the gland. The large-cell neuroendocrine component comprised 70% of the tumor and the acinar adenocarcinoma component accounted for the remaining 30%. Gleason score of the adenocarcinomatous component was 7(3 + 4). There was capsular infiltration at the base of the right lobe. The bladder neck and seminal vesicles were invaded as well as the neurovascular bands. There was also perineural invasion and vascular embolus (Fig. 2). The large-cell neuroendocrine component was confirmed by immunohistochemistry, with labeling by synaptophysin and chromogranin A, with a strong proliferative activity, as Ki67 expression was at 80% (Fig. 3).

All dissected lymph nodes on the right side were negative, and one lymph node of the seven taken on the left side was metastatic, showing invasion by the large-cell neuroendocrine component. The tumor was then classified as pT4 N1 (1/13) R1. Postoperative PSA was 0.01 ng/ml. Following a multidisciplinary medical consultation, it was decided to treat the patient with chemotherapy (etoposide carboplatin [VP16]) as an adjuvant treatment, followed by radiotherapy of the prostatic bed and pelvis.

A [^18^F]FDG PET/CT scan performed in September 2016 showed the absence of any hyper-metabolic site.

Chemotherapy with etoposide carboplatin (VP16) was started in February 2016, then interrupted in May 2016 following the second cycle due to renal toxicity.

The [^18^F]FDG PET/CT scan performed in June 2016, 6 months following surgery, showed two left latero-aortic hypermetabolic lymph nodes despite a PSA of 0.01 ng/ml. The [^18^F]FDG PET/CT scan in August 2016, 4 months after the end of chemotherapy, showed a significant decrease in the metabolic character of the left latero-aortic infracentimetric lymph node and the absence of a second suspicious hypermetabolic lesion; PSA was 0.01 ng/ml. Intensity-modulated radiation therapy (IMRT; 6 MV photons) of the prostatic bed was performed between November and December 2016, with 70 Gy, given in 35 sessions.

The follow-up was then planned based on the PSA level for the adenocarcinoma component and the [^18^F]FDG PET/CT findings for the large-cell neuroendocrine component. In July 2017 the patient presented with an elevated PSA of 2.75 ng/ml; in November 2017 the PSA was 4. 77 ng/ml. The [^18^F]FDG PET/CT scan in November 2017 showed bilateral supracentimetric iliac lymph nodes, poorly hypermetabolic, that were more indicative of the adenocarcinomatous component. Androgen deprivation therapy with the luteinizing hormone-releasing hormone (LHRH) agonist triptorelin was started in December 2017. The biological response was favorable; the PSA was 0.01 ng/ml and testosterone was 0.2 ng/ml 3 months after the first injection of triptorelin. The [^18^F]FDG PET/CT scan in June 2018, 6 months after the initiation of androgen deprivation therapy, indicated a complete metabolic response.

The follow-up was biological, with PSA measurement every 3 months for the adenocarcinomatous component and metabolic imaging with [^18^F]FDG PET/CT every 6 months for the large-cell neuroendocrine component. Currently, after 54 months of follow-up, the patient is still receiving androgen deprivation therapy with triptorelin with complete biological and metabolic response. At the last check-up in March 2020, PSA was 0.01 ng/ml and the [^18^F]FDG PET/CT scan showed a complete metabolic response.

## Discussion

The first descriptions of neuroendocrine system cells are relatively recent, dating from the middle of the twentieth century. Neuroendocrine cells are usually present in prostate tissue and increasing in number after puberty. They are less frequent in acini, from which conventional adenocarcinoma of the prostate can develop. Neuroendocrine cells play a role in the growth and secretory functions of prostatic epithelium [3], and they are recognizable by the absence of androgen receptors and through labeling by certain immunohistochemical markers, such as chromogranin and synaptophysin. PSA negativity in immunohistochemistry is a fundamental criterion of neuroendocrine cell recognition [4]. It is important to note that a focal expression of PSA can be observed within neuroendocrine tumors and that high-grade acinar adenocarcinoma can also express neuroendocrine markers, thus enabling differential diagnosis with large-cell neuroendocrine tumors [2].

The incidence of neuroendocrine cells in conventional prostatic adenocarcinoma varies in the different studies that have histologically assessed primitive tumors and metastases [5].

Focal neuroendocrine features in prostatic adenocarcinoma are correlated to high-grade and undifferentiated tumors. As adenocarcinomatous and neuroendocrine cells can share hybrid immunohistochemical features in some cases, it is important to consider the classical morphological aspects of neuroendocrine tumor cells. Large neuroendocrine cancer cells are usually in large nests with peripheral palisading [6].

Heterogeneity in defining the neuroendocrine status in prostatic tumors can also be related to the increase in life expectancy and the awareness in detecting focal neuroendocrine foci in prostatic adenocarcinoma. Therapies based on androgen receptor signaling inhibition may also interfere with the detection of neuroendocrine features, as in the case of metastases biopsy [7, 8].

Two mechanisms are described in the pathogenesis of prostate neuroendocrine tumors. The first, most frequent, is the transdifferentiation of an acinar adenocarcinoma under long-term hormone therapy, with loss of androgen receptors. Neuroendocrine manifestations of prostate cancer are increasing because of the prolongation of survival and the use of new hormone therapies. The second mechanism is a malignant transformation of neuroendocrine cells usually present in prostatic glands [9]. This mechanism of de novo tumors is supported by carcinogenesis animal models of prostate neuroendocrine cells [10].

Given the limited number of published cases and series, the risk factors predisposing to the development of de novo neuroendocrine tumors has not been established [2]. The 2016 histological classification of tumors of the urinary and genital system described three entities of prostate neuroendocrine carcinoma: neuroendocrine differentiation of adenocarcinoma, well differentiated neuroendocrine tumors or carcinoid tumors and poorly differentiated neuroendocrine small- or large-cell tumors. The recognition of neuroendocrine tumors rests on morphological, functional and immunohistochemical criteria [1].

Neuroendocrine tumors differ from adenocarcinoma by the absence of PSA secretion, resistance to hormone therapy, early metastasis and rapid progression [4]. Small-cell tumors are by far more frequent than large-cell tumors which remain exceptional. The coexistence of the two forms within the same tumor can be observed [4].

Large-cell de novo tumors are rare. The most recent review of the literature, published in 2019, lists only around 20 cases, of which 17 are about primitive prostate tumors, including seven de novo and eight following hormone therapy. Among the cases of large-cell de novo tumors, only three observations report mixed tumors with two components, neuroendocrine and adenocarcinomatous. These three cases responded favorably to hormone therapy [2]. De novo neuroendocrine tumors, either small or large cells, associated with an adenocarcinomatous component seem to have a better prognosis than pure forms because of a certain degree of hormone sensitivity. This notion of androgen dependence or resistance determines the difference in prognosis between the two forms [11]. Because of PSA secretion, mixed forms are susceptible to early diagnosis at the localized stage, providing thus the possibility of curative treatment.

Pure forms, not secreting PSA, are often diagnosed at an advanced stage.

Xiang Tu *et al.* report three cases of de novo large-cell pure neuroendocrine tumors, diagnosed following trans-urethral resection of the prostate, with an unfavorable course under chemotherapy [2]. Evans *et al.* report a similar case with a tumor classified pT3a after radical prostatectomy, with the patient presentinglocal and metastatic cerebral recurrence under adjuvant chemotherapy [5].

Therapeutic possibilities are similar to thosee of lung large-cell neuroendocrine tumors and essentially involves chemotherapy given the hormone therapy resistance. The prognosis is usually unfavorable in the locally advanced and metastatic stages, with a very limited survival [12, 13].

The case we report was diagnosed at a locally advanced stage following ultrasound-guided biopsies. Considering the rarity of the histological type and the moderate renal failure that limited the possibilities of subsequent chemotherapy, we opted for radical prostatectomy rather than radiotherapy. [^18^]F-FDG PET/CT follow-up was justified for a better detection of neuroendocrine cell metabolism, in comparison with choline. The favorable and complete response to hormone therapy, after ganglionic recurrence, can be explained by the presence of an adenocarcinomatous component coexisting with the neuroendocrine component within the same tumor. Complete remission and relatively long survival in comparison with the previously published cases strongly depend on early diagnosis, curative initial surgical treatment and also regular imaging during the follow-up allowing an early detection and adapted treatment of lymph nodes recurrence.

## Conclusions

Large-cell primary neuroendocrine carcinoma of the prostate is an aggressive and a rare histological entity. Curative surgery should be envisaged in localized and locally advanced forms. Hormone resistance in the pure forms limits the therapeutic arsenal for metastatic forms. The association with a hormone-sensitive adenocarcinoma component improves prognosis. The development of nuclear imaging modalities allows a better follow-up and early diagnosis of recurrence, with substantial optimized care.

## Data Availability

The authors make readily reproducible and freely available the materials described in the manuscript to any scientist wishing to use them, without breaching confidentiality. The authors make the materials described in the manuscript available for testing by reviewers in a way that preserves the reviewers’ anonymity.

## References

[1] Humphrey PA, Moch H, Cubilla AL, Ulbright TM, Reuter VE. The 2016 WHO classification of tumours of the urinary system and male genital organs-part b: prostate and bladder tumours. Eur Urol. 2016;70(1):106–19. 10.1016/j.eururo.2016.02.028 26996659

[2] Tu X, Chang T, Nie L, *et al.* Large cell neuroendocrine carcinoma of the prostate: a systematic review and pooled analysis. Urol Int. 2019;103(4):383–90. 10.1159/000499883 30965328

[3] Abrahamsson PA. Neuroendocrine differentiation in prostatic carcinoma. Prostate. 1999;39:135–48. 10.1002/(sici)1097-0045(19990501)39:2<135::aid-pros9>3.0.co;2-s 10221570

[4] Bellur S, Van der Kwast T, Mete O. Evolving concepts in prostatic neuroendocrine manifestations: from focal divergent differentiation to amphicrine carcinoma. Hum Pathol. 2019;85:313–27. 10.1016/j.humpath.2018.11.016 30481509

[5] Fine S. Neuroendocrine tumors of the prostate. Mod Pathol. 2018;31:122–32. 10.1038/modpathol.2017.164 29297494

[6] Epstein JI, Amin MB, Beltran H, *et al.* Proposed morphologic classification of prostate cancer with neuroendocrine differentiation. Am J Surg Pathol. 2014;38(6):756–67. 10.1097/PAS.0000000000000208 PMC4112087 24705311

[7] de Bono JS, Logothetis CJ, Molina A, *et al.* Abiraterone and increased survival in metastatic prostate cancer. N Engl JMed. 2011;364:1995–2005. 10.1056/NEJMoa1014618 PMC3471149 21612468

[8] Scher HI, Fizazi K, Saad F, *et al.* Increased survival with enzalutamide in prostate cancer after chemotherapy. N Engl J Med. 2012;367:1187–97. 10.1056/NEJMoa1207506 22894553

[9] Evans AJ, Humphrey PA, Belani J, van der Kwast TH, Srigley JR. Large cell neuroendocrine carcinoma of prostate: a clinicopathologic summary of 7 cases of a rare manifestation of advanced prostate cancer. Am J Surg Pathol. 2006;30:684–93. 10.1097/00000478-200606000-00003 16723845

[10] Garabedin EM, Humphery PA, Gordon JI. A transgenic mouse model of metastatic prostate cancer originating from neuroendocrine cells. Proc Natl Acad Sci USA. 1998;95:15382–7. 10.1073/pnas.95.26.15382 PMC28051 9860977

[11] Priemer DS, Montironi R, Wang L, Williamson SR, Lopez-Beltran A, Cheng L. Neuroendocrine tumors of the prostate: emerging in-sights from molecular data and updates to the 2016 World Health Organization Classification. Endocr Pathol. 2016;27(2):123–35. 10.1007/s12022-016-9421-z 26885643

[12] Tzou KY, Cheng WH, Lee WH, Ho CH. Primary large cell neuroendocrine carcinoma of the prostate in a hormone naive patient: a case report from Taiwan. J Cancer Res Ther. 2018;14(10 Suppl):S785–8. 10.4103/0973-1482.180685 30249904

[13] Okoye E, Choi EK, Divatia M, Miles BJ, Ayala AG, Ro JY. De novo large cell neuroendocrine carcinoma of the prostate gland with pelvic lymph node metastasis: a case report with review of literature. Int J Clin Exp Pathol. 2014;7(12):9061–6. PMC4313989 25674288

